# The Relevance of Online Social Relationships Among the Elderly: How Using the Web Could Enhance Quality of Life?

**DOI:** 10.3389/fpsyg.2020.551862

**Published:** 2020-10-02

**Authors:** Martina Benvenuti, Sara Giovagnoli, Elvis Mazzoni, Pietro Cipresso, Elisa Pedroli, Giuseppe Riva

**Affiliations:** ^1^Istituto per le Tecnologie Didattiche, Consiglio Nazionale delle Ricerche, Genova, Italy; ^2^Dipartimento di Psicologia, ALMA MATER STUDIORUM – Università di Bologna, Cesena, Italy; ^3^Applied Technology for NeuroPsychology Lab, Istituto Auxologico Italiano, Milano, Italy; ^4^Dipartimento di Psicologia, Università Cattolica del Sacro Cuore, Milano, Italy

**Keywords:** elderly, Internet, quality of life, social support, well-being

## Abstract

This observational study analyzes the impact of Internet use on the quality of life and well-being of the elderly. Specifically, it seeks to understand and clarify the effects of Internet use on relationships in terms of self-esteem, life satisfaction, and Online and Offline Social Support in a sample of senior and elderly Italian people (over 60 years of age). A cohort of 271 elderly people (133 males and 138 females) aged between 60 and 94 years old participated in the study: 236 were Internet Users while the other 35 were Non-Internet Users. The results showed that the time elderly people spend online has a negative effect on their perception of Offline Social Support (Offline Emotional and Informational and Offline Affective Social Support) and a positive effect on their perception of Online Social Support (particularly on Online Positive Social Interactions). Surprisingly, Internet use among elderly people seems to positively affect the perception of Offline Social Support. Indeed, elderly Internet Users have a more positive perception of Offline Social Support (particularly Offline Positive Social Interactions and Offline Affective Social Support) than Non-Internet Users. A discussion of this finding is provided, positing that the Internet seems to represent the technological side of a functional organ that allows the elderly to stay in closer touch with their family and friends and in doing so to also overcome some age-related difficulties.

## Introduction

In 2019, around 10% of the world’s population was over 65 years old, and this percentage is expected to increase significantly by 2050. Currently, in the European Union, 18% of the approximately 750 million people are over 65 years, and the age group of the over 80s is increasing faster in Europe than in non-European countries.

The distinction between periods of life or stages of development such as “third age” and “fourth age” is fundamentally arbitrary, and there are no strict criteria that determine the transition from the former to the latter (Titmus, 2014). While in developed countries most “third age” (generally around 65 years) individuals have sufficiently good health conditions that allow them to live independently in private homes and participate actively in social life, the “fourth age” (around the age of 85 years) refers to people who mainly experience difficulties in managing with independent living; they often need medical treatment and become fundamentally dependent on one or more caregivers (Stephens et al., 2015). Indeed, in developed countries, it is evident that people over the age of 85 are the most susceptible to disease and disability (Unger, 2012). Physiological aging impacts sensorial perception (hearing, vision, taste, and proprioception), the motor system (due to neuronal decay and/or loss of muscle tone), as well as cognitive abilities. This places an unavoidable economic burden on health services, a factor that should be appropriately considered when assessing the sustainability of social systems of the future (Sharkey and Sharkey, 2012). However, it should also be remembered that physical–cognitive decay through aging produces a spiraling process that makes it increasingly difficult for the elderly to remain physically mobile; given that they are already affected by the progressive thinning of social networks due to mortality, the elderly run the risk of progressive isolation from social life (Rosso et al., 2013).

For this reason, Stephens et al. (2015) affirm that in addition to the design of intervention programs aimed primarily at individual wellness, it is useful to deal with areas related to the management of the social and physical environment of reference of the elderly; this could help alleviate much loneliness and social exclusion. In this regard, caregivers need access to adequate tools and strategies for encouraging the maintenance and creation of social support. Among the principal aspects to consider for promoting good health in the elderly and planning useful prevention strategies to improve their quality of life (QoL), Foster and Walker (2015) suggest:

– The role and effectiveness of the network of social relationships in support of the elderly, and strategies for maintaining good levels of autonomy and independence even in advanced age;
– The role of technological aids in reducing disability and alleviating functional decline in the elderly, particularly via technological innovations aimed at improving communication, tecno-assistance, and home care.

Therefore, the individual’s social and community context as part of a dynamic system plays a fundamental role in defining the general state of health of the person: well-being is a social construct and, as such, finds expression also in the capital of human relations (Marmot et al., 2012). As highlighted above, the maintenance and enhancement of social networks are relevant aspects for so-called Active Aging (Fernández-Ballesteros, 2008); with advancing age, the risk of progressive weakening and loss of contacts and bonds increases, and this can lead to situations of exclusion and social isolation. Given the growing interest in the potential of social relationships networks as a means for promoting the mental health and well-being of the elderly, the concept of social capital has become increasingly relevant (World Health Organization, 2013).

## Social Capital, Social Support, and ICT for Promoting QoL Among the Elderly

QoL is a multidimensional concept that embraces subjective and objective aspects of a person’s life situation contextualized in a given socio-cultural and economic environment (Phillips et al., 2010), with particularly strong social connotations. Indeed, as widely documented in the literature, the concept of health is closely related to the social resources available to the individual. Therefore, social capital in advanced age can be considered a fundamental component in determining and defining the state of well-being and QoL (Nyqvist et al., 2013). The functional dimension of social capital is social support, which represents a fundamental element of QoL among the elderly, manifesting in emotional, instrumental, informative, and evaluation support (Phillips et al., 2010).

Bourdieu (1980) introduced the concept of social capital, defining it as the set of resources that are embedded in networks of relationships and which can be accessed in case of need.

The concept of social capital can be defined and measured through different dimensions, which include indicators such as the quantity and quality of social relations, and that determine different types of relations, e.g., bonding and bridging ties (Poortinga, 2012). Bonding social capital, like the relationships that characterize the family context, refers to strong intra-group bonds, while bridging social capital refers to the construction of weaker bonds between heterogeneous groups. Strong ties are not very diversified in terms of social extraction but offer relationships characterized by greater interpersonal closeness (Williams, 2006). Bridging ties include those between acquaintances and are established among more diverse and heterogeneous groups. These links are more cross-sectoral than strong ties and provide access to different and more varied resources (Granovetter, 1973, 1982) or other information that is not available in a close social network (typified by the sharing of largely common information, knowledge, and practices).

While in the past social capital was based on face-to-face interactions, phone calls, or postal correspondence, nowadays a large part of human intercommunications are conducted via ICT and Internet tools. Currently, these mostly involve the use of computers, tablets, and especially smartphones to access online meeting places and synchronous and asynchronous communication channels [e-mail, audio-video conferencing systems, chat systems, social networking sites (SNSs), etc.]. However, what possible role can digital devices play in creating or maintaining networks of relationships related to social support? Moreover, could the use of ICT, therefore, particularly in advanced age, contribute to the promotion of active and healthy aging?

In the case of bonding social capital and the Internet, few studies have examined the relationship between the Internet and bonding social capital (Williams, 2006). Although scholars have envisaged a probable net loss of social capital in face-to-face interactions as a result of growing usage of the Internet, Williams suggests that many studies have failed to focus on the benefits that online interactions could yield in replacing, maintaining, or integrating strong networks. Indeed, most studies that investigate the effects of using the Internet focus on the associated risks (Mazzoni et al., 2016). Dwelling exclusively on the potentially harmful aspects deriving from the use of the Internet can however, be reductive and misleading. Instead, a positive approach to the study of the effects that technology has on real life allows us to consider the potential resources that Internet access can grant access to, even the case of quite elderly users. For example, an exploratory study of over-sixty Internet Users in Australia (Russell et al., 2008) has shown that although the Internet is not the only tool capable of maintaining social networks in advanced age, its use can bolster real social capital, preventing the loss of previously established contacts.

From the Vygotskian concept of the Zone of Proximal Development and that of functional artifacts developed by Leontev (1978), Internet tools could be seen as functional organs (Kaptelinin, 1996) that allow people to achieve better performance than they would without technological devices; in case of elderly people, they can compensate for weakness or loss of individual or structural capacities due to aging. However, in some cases, digital tools are used not for their primary function but rather to compensate for something lacking in real life, such as taking pictures not to record and share an event or experience but rather to capture general attention, receive likes, and enhance one’s self-esteem. In such cases, there is a risk that instead of making constructive use of the tool, the user loses control of it and becomes subordinate to it, objectifying any potential active role. Ekbia and Nardi (2012) name this process *inverse instrumentality*, since the user risks losing control of the objectives of their actions this would happen, for example, if the user were to attend a cultural event, such as a concert, with the primary intent of taking pictures to upload online so as to boost self-esteem, rather than to appreciate the artists’ performance. So, to understand if and how technologies can influence the creation and maintenance of social capital relationships, it is necessary to investigate what factors and conditions incline the user toward use of technology as a functional organ or instead in a condition of inverse instrumentality (Frozzi and Mazzoni, 2011).

Regarding the role that the use of technologies can play in maintaining or creating social networks, Zywica and Danowski (2008) have observed how people who have more well-developed social networks offline tend to grow and expand their network of relationships through online interactions, as a sort of enhancement of the former. Indeed, some studies (Lampe et al., 2006; Lenhart and Madden, 2007) suggest that SNSs are frequently used to stay in touch with people that are part of a person’s offline, or real, social capital. Results from these studies show, for example, that Facebook users are also (or even more) interested in connecting with people they already know in the real world rather than exclusively online connections. Boyd and Ellison (2007) stressed that, prior to the diffusion of SNSs, offline and online communities were largely disconnected, with little of the overlap we see today. Although these results come from studies carried out with teenagers and young people, we can hypothesize that the same is even more evident for elderly people who, already having a structured social capital, do not have the same need to expand their existing social networks as they have more chances to stay in touch with them.

However, even people with weaker and less developed offline social networks tend to expand their online relationships, possibly as a compensatory effect. By following the “social compensation vs enhancement hypothesis” of Zywica and Danowski (2008); Mazzoni et al. (2016) showed how the Internet can be used as a functional organ in the creation and maintenance of the capital of relationships where there is already a good level of Offline Social Support in real life. When, on the other hand, offline social networks are weak or inexistent, a compensatory mechanism could be triggered to try online to fill this gap, thus running the risk of inverse instrumentality.

Unfortunately, the role of Information and Communication Technologies (ICT) in determining the QoL of elderly people through the creation and maintenance of social capital has generally been neglected in social gerontology, with existing studies mainly focusing on medical and care technologies (Xie, 2007).

An interesting review by Boz and Karatas (2015) considered 25 studies from the period 2000–2015, examining the impact of Internet use on elderly people’s QoL. The review demonstrated that functional use of the computer and Internet can improve the life quality of the elderly with 15 of the 25 studies showing a significant relationship between Internet use and QoL for the elderly, whereas no relationship was found in other studies.

In one of the earliest studies investigating the effect of Internet use on social relations and QoL among the elderly, White et al. (1999) provided Internet and e-mail access to senior residents at a retirement center. A significant decrease in the loneliness and social isolation level of the elderly was found in the intervention group, while in the comparison group the same factors remained unchanged. In a more recent study (Osman et al., 2005), 50 elderly people were provided with a computer with Internet and access to a web portal specifically addressing the needs of the elderly; participants received thorough training on Internet use throughout the project. During the post-intervention interviews, the participants described Internet use as a potential “window on the outside world.” During the course of the study, it was observed that access to the Internet and to e-mail reduced the social isolation of the elderly participants and strengthened their social interactions.

Even more recently, Sum et al. (2008) explored the impact of Internet use on the sense of belonging to a community in advanced age, showing that it facilitates the elderly’s social interaction with family members and friends and extends their social network. The results of this study show the complex relationship between use of the Internet, social capital, and well-being; being connected online influences the social capital and well-being of elderly people in different ways, partly in relation to the average time spent on online activities. Going into detail, the authors observed that where the Internet is used to search for information and to communicate with others (as correlated with high levels of well-being and lower levels of perceived loneliness), a positive impact on social capital is generated. By contrast, Internet use to meet new people and pass the time (as related to lower levels of well-being and greater perception of loneliness) has a significantly lower impact on the creation of social support networks. These results, therefore, underline that the use of technologies to compensate for the lack of satisfying and meaningful relationships in real life can lead to situations of inverse instrumentality, while the technological tool becomes a functional organ when it is a support for an existing network of offline relationships (Sum et al., 2008; Mazzoni et al., 2015).

An interesting study, based on the European Social Survey (ESS1), examined the impact of Internet use on social isolation and well-being of 11,000 Internet Users aged 65 from 26 countries (Lelkes, 2013). As the results indicate, the elderly’s face-to-face interactions are not replaced by online communication, which, on the contrary, acts as a complementary and strengthening factor for the former. Indeed, even though the subjects communicated with their family and friends over the Internet, they reported that they still prefer to meet people face to face. Furthermore, the results showed that elderly people who use the Internet on average for more time during the day tend to have lower chances of meeting face to face with the people who belong to their social capital.

A recent Korean study (Nam, 2019) examined the mediating effect of social support on the relationship between older adults’ use of social media and their QoL. The results confirmed the mediating effect of social support and showed that social media use not only had a direct effect on QoL but also had an indirect effect through social support. However, another study (Kim, 2018) with a similar sample (Korean adults aged 65 and over) examined the influence of smartphone use on life satisfaction, depression, social activity, and social support and found different results. Specifically, smartphone use produced no statistically significant impact on older adults’ social support, even though the general results of the study suggest that use of ICT such as smartphones in advanced age can play generally positive roles in enhancing psychological, mental, and social aspects of the QoL.

The inverse proportionality between Internet use and QoL (particularly as it pertains to social contacts) has also been highlighted by Khalaila and Vitman-Schorr (2018). In their analysis of the extant literature, they show that Internet use is significantly associated with decreased time spent with friends and decreased local social networking, with a risk of increasing loneliness and decreasing various aspects of QoL (Kraut et al., 1998; Coget et al., 2002). Indeed, Internet use would seem to not only replace face-to-face contacts with weaker online ties but also diminish participation in in-person social activities (Nie and Hillygus, 2002), thus outing personal contacts at risk (Veena et al., 2012) and generating new forms of isolation and marginalization (Gardner et al., 2012). However, on the other hand, many other studies suggest that older adults can benefit from Internet use in many aspects of their social life, such as constructing new personal friendships, enhancing the quantity and quality of contacts with family and friends, and maintaining social involvement (Veena et al., 2012; Chen and Schulz, 2016; Yu et al., 2016). In this light, Khalaila and Vitman-Schorr (2018) explored the direct and/or indirect effects of Internet use on QoL, by conducting structured interviews with a sample of 502 respondents aged 50 and older living in northern Israel. Their results show that Internet use is positively associated with QoL, with loneliness being a mediation effect: Internet use is associated with a lower level of loneliness, which in turn is related to greater QoL. Furthermore, results show a moderated effect of time spent daily with family members: Internet use seems to be helpful in increasing QoL only for those who spend average or above-average time with their family. Since without face-to-face contacts Internet use does not represent an added value on predicting QoL, the authors’ interpretation is that online contacts (Internet use) and face-to-face contacts (time spent with family) have a synergistic effect on QoL.

In a study with a sample of adults living in Portugal, of which 118 were older adults, Neves et al. (2018) found that social capital is positively related to Internet use but negatively related to age. On the one hand, greater Internet use increases the likelihood of having a high level of social capital. On the other hand, the Internet seems to counterbalance the negative relationship between age and social capital: indeed, older heavy Internet Users were more likely to have high levels of social capital than older light-users or non-users. However, only more highly educated older adults seem to profit from that buffering moderating effect of Internet use. In addition to these results, this study is very interesting since it proposes a difference between elderly users, elderly light-users, and elderly non-users. Furthermore, it considers both online and offline social capital, as they each could have a different and interactive effect on elderly QoL. Interestingly, to measure social capital, the authors proposed a combination of indicators to capture quality and quantity of social ties. In particular, they also used items taken from the Internet Social Capital Scales (Williams, 2006), which consider offline and online bonding and bridging dimensions and feature items very similar to those adopted in the more recent Offline/Online Social Support Scale (Leung and Lee, 2005; Wang and Wang, 2013). Finally, as the authors claimed, the Internet seems to play a contrasting role in the life of the elderly. While it helps them to accrue, maintain, and mobilize social capital, its cumulative advantage effect could reinforce forms of social inequality, particularly those determined by age and education.

## The Study

Starting from the extant literature, this observational study considers social capital (both offline and online) as a significant resource for promoting well-being and QoL among elderly people (Nyqvist et al., 2013). Thus, considering social capital as the social ties that could support older people in their daily life in both offline and online contexts (Wang and Wang, 2013; Mazzoni et al., 2016), this study seeks to understand and clarify the effects of Internet use on relationships that characterize self-esteem, life satisfaction, and Online and Offline Social Support in a sample of elderly Italian people (over 60 years). Indeed, as described earlier and as reported by several studies on Active Aging (Boudiny, 2013; Foster and Walker, 2015), self-esteem and life satisfaction are seen as fundamental dimensions in determining the levels of well-being of the person: more precisely, results show a general decrease in the levels of self-esteem and satisfaction with one’s life with advancing age (McAuley et al., 2000; Robins et al., 2002; Pavot and Diener, 2008). At the same time, in the light of the literature reported herein, given that all these factors are related to the QoL and well-being, the study also intends to understand which of the considered Internet use factors prove particularly significant in determining QoL and well-being of the elderly.

## Materials and Methods

### Recruitment Strategy and Data Collection

Recruiting the participant sample for the study was a particularly delicate process. First of all, an online search led to identification of organizations, associations, and centers for lifelong learning that hold digital literacy courses for the elderly; these were then contacted to propose subject participation in the study. One particularly interesting recruitment source was a non-profit organization offering a third-age digital literacy program to train elderly people to use the web and promote active aging. Other members of the subject cohort included participants in computer literacy courses for over-65s that were held by a local council in central Italy. Finally, participants in evening digital literacy courses for over-60s held by a cultural association in a small town in central Italy also agreed to participate. In each context, during the first training session of the respective courses, all participants were required to fill in an online questionnaire with the support of tutor-trainers where needed.

The first part of the questionnaire concerned informed consent about participation in the study and data treatment. Even though the adopted sampling procedure can be seen as a limitation of the study, it allowed us to reach a large number of elderly people with a common interest in learning how to use digital technologies, albeit from different competence/experience baselines.

The online questionnaire was structured in three parts:

• Sociodemographic information (gender, age, region, and province of residence, educational level, current employment, and housing status).
• Information related to access and use of digital technologies, specifically type of device (PC, tablet, and smartphone); average daily hours of use of each device; how access to the device came about and how the subject learned to use it; frequency of activities carried out online; frequency of online interactions with various connection types (weak and strong ties); willingness and interest in improving skills in using technologies to carry out different online activities.
• Information regarding those parameters that the extant literature identifies as influencing QoL: social support, self-esteem, and life satisfaction. This last one was measured using validated scales.

### Measures

#### Offline and Online Social Support

An Italian translation of the Offline and Online Social Support Scales (Leung and Lee, 2005; Wang and Wang, 2013) for the measurement of social support perceived in both offline and online relationships was adopted. Both scales consist of 11 items related to the initial question “How often is each of the following kinds of support available to you if you need it?” To answer, participants select a response from one of the five levels in which the proposed Likert scale response range is divided (from never to always). The scale items can be divided into three subscales (Emotional and Informational, Positive Social Interaction, and Affective). Items 1, 2, 3, and 4 refer to Emotional and Informational (EI) social support, which involves caring, warmth and affection, and sympathy, as well as offering guidance, advice, information, or feedback for problem solving. Items 5, 6, and 7 refer to Positive Social Interactions (PSI), also called social companionship, as it is related to spending time with others in leisure and recreational activities. Finally, items 8, 9, 10, and 11 refer to Affective (AF) social support characterized by (non-problem related) expressions of love and affection.

#### Self-Esteem

To collect data about self-esteem, the Italian adaptation (Prezza et al., 1997) of the Rosenberg Self-Esteem Scale (Rosenberg, 1965) was used.

#### Life Satisfaction

To measure participants’ general life satisfaction and perceived subjective well-being, we used the Italian adaptation (Di Fabio and Gori, 2016) of the Diener Satisfaction with Life Scale (Diener et al., 1985). To answer the five items in the scale, participants selected from seven levels on a Likert scale (from Strongly disagree to Strongly agree).

#### Variables Considered for the Analyses

Based on what has been previously described, analyses have been focused particularly on the following variables:

• Gender.
• Age.
• Type of devices (computer, tablet, smartphone, more than one device).
• Time spent online.
• Offline Social Support (OffLine SS): Emotional and Informational (OffLine EI); Positive Social Interactions (OffLine PSI); Affective (OffLine AF).
• Online Social Support (OnLine SS): Emotional and Informational (OnLine EI); Positive Social Interactions (OnLine PSI); Affective (OnLine AF).
• Rosenberg Self-Esteem.
• Life Satisfaction.

### Description and Organization of Sample

The sample was composed of 271 subjects (133 males and 138 females) aged between 60 and 94 (mean = 72.48; SD = 6.07) who were attending one of the digital literacy courses previously described. In terms of household status, most of the participants stated that they live with a spouse/partner (71.2%), 8.1% with another family member (8.1%), while 20.7% lived alone. The vast majority of the sample (88.9%) were pensioners, and their overall educational level was high by Italian standards for this demographic (52.8% had at least gained their high school diploma).

The total sample was divided into two subgroups: Internet Users (IU) and Internet Non-Users (INU). The IU group comprised 236 participants (118 males and 118 females, mean age = 72.33, SD = 6.14), while the INU group totaled 35 subjects (20 males and 15 females, mean age = 73.49, SD = 5.57).

Subjects belonging to the IU group were subsequently divided into three subgroups on the basis of their habitual device use. These were the PC group (61 participants who only access the Internet using a computer or tablet), the SP group (smartphone Internet Users), and the MD group (141 subjects who access the Internet using multiple device types).

A complete statistical breakdown of the whole sample and of the subgroups is presented in Table 1, showing participant’s age, gender, household status, occupational status, and education level.

**TABLE 1:** Descriptive statistics of the total sample and the subgroups (IU and INU) according to the participant’s age, participant’s sex, housing status, occupation, and education level.

	IU (*n* = 236)	INU (*n* = 35)	Overall (*n* = 271)
Age (mean ± SD)	72.33 ± 6.14	73.49 ± 5.57	72.48 ± 6.07
**Gender (*n*)**			
Males	118	20	133
Females	118	15	138
**Housing status (*n*)**			
Alone	47	9	56
Spouse/partner	173	20	193
Other family members	16	6	22
**Occupation (*n*)**			
Employed	13	2	15
Retired	212	29	241
Unemployed	11	4	15
**Education level (*n*)**			
Low-level education	109	19	128
High-level education	127	16	143

*IU, Internet Users group; INU, Internet Non-Users group.*

### Statistical Analysis Strategy

First, a one-way multivariate analysis of covariance (MANCOVA) was performed on Offline Social Support and its subscales (EI, PSI, and AF), Online Social Support and its subscales (EI, PSI and AF), the Rosenberg Self-Esteem Scale, and the Satisfaction With Life Scale, namely, the dependent variables of interest in the study. GROUP (PC, SP, MD) was used as a between-subject factor, while TIME (total hours spent online) and AGE were treated as covariates.

A second one-way MANCOVA was performed on the Offline Social Support scale and its subscales, on the Rosenberg Self-Esteem Scale, and on the Satisfaction With Life Scale (dependent variables), while GROUP (IU vs INU) was treated as a between-subject factor and AGE was treated as a covariate.

The MANCOVA assumptions (multivariate normality, homogeneity of variances, equal covariance matrices across groups, and uncorrelated model errors) were carefully checked and were met.

## Results

The one-way MANCOVA showed no significant differences between groups [GROUP effect: *F*_(__9_,_218__)_ = 1.61; *p* = 0.053; partial η^2^ = 0.06]. More specifically, the groups did not differ for Offline and Online Social Support scales and subscales, for Rosenberg Self-Esteem level, or for life satisfaction (see Additional Table S1 in the Supplementary Material for detailed results).

A significant TIME effect was found [*F*_(__9_,_218__)_ = 3.19; *p* = 0.001; partial η^2^ = 0.12]; time spent online significantly predicted the majority of the dependent variables included in the analysis (see Table 2). Time spent online negatively affected perceived Offline Social Support (SS), perception of Offline Affective support (AF), and perception of Emotional and Informational support (EI; see Table 2). A significant positive TIME effect was found on the Online Social Support (SS) perception and on perceived Online Positive Social Interaction (PSI; see Table 2).

**TABLE 2:** One-way MANCOVA results: parameter estimates table of TIME effect on OffLine scale and subscales, OnLine scale and subscales, Rosenberg Self-Esteem Scale, and Life Satisfaction Scale.

**Parameter: TIME**				

Dependent variables	*b*	*SE*	*t*	Partial η*^2^*
OffLine SS	–0.02	0.01	−2.33**	0.02
OffLine EI	–0.02	0.01	−2.17**	0.02
OffLine PSI	–0.00	0.01	–0.280	0.00
OffLine AF	–0.03	0.01	−2.93**	0.04
OnLine SS	0.02	0.01	2.15**	0.02
OnLine EI	0.01	0.01	1.28	0.01
OnLine PSI	0.03	0.01	3.11**	0.04
OnLine AF	0.02	0.01	1.86	0.02
Rosenberg Self-Esteem	–0.07	0.03	−2.63**	0.03
Life Satisfaction	–0.04	0.01	−2.59**	0.03

EI, Emotional and Informational support; PSI, Positive Social Interaction; AF, Affective Support; SS, Social Support. *p < 0.05; **p < 0.01.

These results indicate that the more the subject uses the Internet, the lower their perception of Offline Social Support. On the contrary, greater Internet use raises the perception of Online Social Support.

A significant TIME effect was found regarding self-esteem, in that time spent online negatively predicts the subject’s self-esteem. A similar result was found for the Life Satisfaction Scale: time spent online negatively predicts the subject’s life satisfaction (see Table 2).

AGE was not found to have a significant transversal effect [*F*_(__9_,_218__)_ = 1.74; *p* = 0.08; partial η^2^ = 0.07]. However, univariate analysis did show a significant AGE effect on the perception of positive online social interaction (PSI), showing how age negatively predict online PSI (Parameter estimates: *b* = -0.03, SE = 0.01; *p* = 0.04; partial η^2^ = 0.02).

One-way MANCOVA applied to compare Internet Users (IU) and Internet Non-Users (INU) showed a significant GROUP effect [*F*_(__6_,_256__)_ = 2.23; *p* = 0.04; partial η^2^ = 0.05]. As Table 3 shows, the INU group perceived a general lower Offline Social Support compared to the IU group [*F*_(__1_,_261__)_ = 4.43; *p* = 0.04; partial η^2^ = 0.02]. A lower Positive Social Interaction [*F*_(__1_,_261__)_ = 4.54; *p* = 0.03; partial η^2^ = 0.02] and a lower Affective Social Support [*F*_(__1_,_261__)_ = 6.21; *p* = 0.01; partial η^2^ = 0.02] compared to IU groups was also found (see Table 3).

**TABLE 3:** Estimated marginal means on OffLine scale and subscales, Rosenberg Self-Esteem Scale, and Life Satisfaction Scale in Internet Users group (IU) and Internet Non-Users group (INU).

	IU; *n* = 231 (mean ± SE)	INU; *n* = 33 (mean ± SE)
OffLine SS	3.370.07	3.200.18
OffLine EI	3.520.0	3.150.16
OffLine PSI	3.880.07	3.420.17
OffLine AF	3.580.05	3.250.14
Rosenberg Self-Esteem	22.550.19	22.340.51
Life Satisfaction	5.030.10	5.250.26

*Covariates appearing in the model are evaluated at the following values: AGE = 72.43. EI, Emotional and Informational support; PSI, Positive Social Interaction; AF, Affective Support; SS, Social Support.*

No significant difference on self-esteem level [*F*_(__1_,_261__)_ = 0.15; *p* = 0.70; partial η^2^ = 0.0] or on life satisfaction [*F*_(__1_,_261__)_ = 0.61; *p* = 0.44; partial η^2^ = 0.0] was found.

Results showed a significant general AGE effect [*F*_(__6_,_256__)_ = 2.82; *p* = 0.01; partial η^2^ = 0.06]. Looking at the estimated parameters, it emerged that age is a significant predictor only of the perception of Positive Social Interaction; in particular, with increasing age, the perception of Positive Social Interaction decreases (*b* = -0.02, SE = 0.01; *t* = 0.22.28, *p* = 0.02; partial η^2^ = 0.02).

## Discussion

In general, three main results emerge from the data analysis. First, the amount of time elderly people spend online affects their self-esteem and life satisfaction. Second, the same independent variable affects their perception of Social Support, both of the Offline and Online varieties. Finally, elderly people’s Internet use is correlated to their perception of Offline Social Support.

The first result could be interpreted in two different ways depending on the adopted perspective. Following Neves et al. (2018), and also the literature analysis of Khalaila and Vitman-Schorr (2018), we could say that elderly people’s Internet use seems to have a cumulative disadvantage effect: the more they use the Internet, the more this could decrease their autonomous movement and opportunities to engage in face-to-face contacts. These factors would present the risk of isolation, thereby negatively affecting their self-esteem and life satisfaction. However, following the social compensation hypothesis of Zywica and Danowski (2008), the lower elderly people’s self-esteem and life satisfaction (perhaps related to low levels of health, autonomy, social contacts, etc.), the more they try to compensate offline life deficiencies via online experience. Thus, they tend to pass more time on the Internet to try to satisfy their needs.

Although the other two results regarding social support are both very interesting, the first is not so surprising as the second. The first shows a double effect of time of Internet connection on the perception of Social Support: negative for the Offline variety and positive for Online. This result can be interpreted in the light of the study by Lelkes (2013) in which older people reported preferring to meet their family and friends face to face than communicating with them over the Internet. A hypothesis that Turkle formulated (Turkle, 2017) is that when people pass a lot of time online, they end up preferring online relations to offline ones, due to the illusion of control over interactions that Internet applications (particularly chats) can generate. Thus, despite the functional effect for Online Social Support, elderly people passing more time on the Internet seems to have an inverse instrumental effect (Ekbia and Nardi, 2012) on Offline Social Support. This is probably because the time spent online leaves them less time for face-to-face interactions, thereby negatively affecting their self-esteem and life satisfaction. The inverse instrumental effect, following the compensation hypothesis of Zywica and Danowski (2008), could be generated by the fact that the more elderly people pass time online, the more they perceive a loss in Offline Social Support, and so the more they tend to compensate this by constructing Online Social Support (or re-construction Offline support in Online contexts).

Interestingly, online time negatively affects Emotional and Informational Offline Social Support and Affective Social Support, while it positively affects Online Positive Social Interactions. Thus, in the perception of elderly people, the Internet seems to be particularly useful for leisure activities with others, but it negatively affects those everyday life relations characterized by caring, warmth and affection, sympathy, and their expression. As the elderly, like most people, prefer to meet their family and friends face to face, and considering the differentiation between bridging and bonding social capital (Putnam, 2001; Poortinga, 2012), a hypothesis could be that the more the elderly are circumstantially compelled to use the Internet to stay in touch, the more they perceive a loss in bonding social capital, notwithstanding a perceived gain in online social companionship (which can be associated to bridging social capital).

The second general result (amount of Internet time affects perception of both Offline and Online Social Support) seems surprising since one could hypothesize, in line with the first result, which elderly people who do not use the Internet have a perception of higher Offline Social Support than those who do. In fact, the study results show exactly the opposite outcome. Before hazarding an interpretation, we should consider that many factors that were outside the scope of this observational study play a role in the use of ICTs and QoL in senior adults, e.g., interest levels and socioeconomic and health requirements. In particular, some health problems can affect Internet use among older adults. As reported in a study by Choi and DiNitto (2013), dementia was categorized as being negatively associated with Internet use, while Internet-related affordances like getting information about chronic health conditions or contacting a medical provider was positively associated. Unfortunately, in the present study, it was not possible to control for all these factors while also respecting the protocols of the participating associations; that said, all the participants reached their various course locations autonomously so we can assume that they enjoy relatively good health levels. An alternative interpretation of this result can be gleaned by rereading it in the light of those studies suggesting that SNSs are frequently used to stay in touch with people that are already part of offline or real, social capital (Lampe et al., 2006; Lenhart and Madden, 2007). A hypothesis is that as many elderly people already have a structured social capital, they do not have such an urgent need to *expand* their existing social networks, but rather to *stay in touch* with them. From this point of view, the Internet becomes part of a functional organ (Kaptelinin, 1996) that allows elderly people to enrich their opportunities to stay in touch with their family and friends or to overcome any difficulties in staying in touch with them. Thus, for elderly people, the Internet can really be interpreted as a supporting technology, helping them to maintain and entertain existing offline social relations more frequently and in diverse communication modalities. Interestingly, the hypothesized effect seems to be particularly useful not only for the perception of social companionship (Positive Social Interaction) but also for the expression of love and affection (Affective Social Support). This is probably due to the fact that the Internet provides the means to stay in touch with those persons (bridging social capital) with whom it can be difficult to stay in frequent contact. Thanks to SNS feedback functions such as *likes* and *follows*, Internet use also simulates responses typical of Offline Affective Social Support.

## Limitations of the Study

The most important limitation of the study is the adopted sampling procedure, which only allowed data collection from about 33 elderly Internet Non-Users. Despite this critical aspect, the recruitment procedure allowed us to reach a large number of elderly people with different degrees of digital experience but with a proactive interest in learning how to use some digital technologies. In future research, a more carefully designed and broadly based sampling procedure could guarantee greater sample balance and the possibility of implementing a quasi-experimental procedure.

A further limitation is that the study considers principally the time-based quantity of Internet use but not the quality, i.e., the actual activities carried out online by elderly people. Even though many previous studies described in the literature show that (excessive) time spent online is one of the most significant factors determining dysfunctional Internet use, future research could nonetheless consider the range of different activities the elderly perform online (e.g., seeking out health information, interacting with friends or with general contacts, playing videogames, etc.). and also the time spent on each one.

## Conclusion

The results from the above reported analysis allow us to affirm that the Internet is a functional and significant technological artifact that is beneficial for the QoL and well-being of elderly people. Indeed, even though passing substantial amounts of time online can have a detrimental effect on perception of Offline Social Support, elderly people who lack an online life miss multiple opportunities to stay in touch with family and friends and have fewer opportunities for companionship. From this point of view, paraphrasing Paracelsus, we could say that “All things are poison and nothing is without poison; only the dose makes a thing not a poison.” For the elderly, the Internet can be a very useful resource to stay in touch and maintain social relationships, whether weak or strong, provided that this is not at the cost of face-to-face meeting spaces, sometimes limited by their own contingencies (for example, limited mobility due to age), but often also by those of family and friends (for various reasons). For these reasons, future research should deepen the relationship between the elderly and the Internet, focusing on motivations for using the Internet. This could reveal whether their Internet use is proactive and employed to exploit its inherent potential or is more simply determined by personal contingencies or those arising from the circle of family, friends, and acquaintances.

## Data Availability Statement

The raw data supporting the conclusions of this article will be made available by the authors, without undue reservation.

## Ethics Statement

The studies involving human participants were reviewed and approved by University of Bologna Bioethics Committee. The patients/participants provided their written informed consent to participate in this study.

## Author Contributions

All authors wrote the manuscript, analyzed the data, interpreted the findings, and reviewed the manuscript. MB and EM designed the current study and recruited the participants.

## Conflict of Interest

The authors declare that the research was conducted in the absence of any commercial or financial relationships that could be construed as a potential conflict of interest.
