# Supplementary material for: The Relevance of Online Social Relationships Among the Elderly: How Using the Web Could Enhance Quality of Life?
Source: Front Psychol. 2020 Oct 2;11:551862. doi: 10.3389/fpsyg.2020.551862 (PMC7566904; doi:10.3389/fpsyg.2020.551862)
Supplement: Supplementary file 1 [file Additional_Table_S1.docx]

| Group effect: F(9,218) = 1.61; p = .053; partial η2 = .06 |  |  |  |  |  |  |
| --- | --- | --- | --- | --- | --- | --- |
| Dependent variables | F(2,226) | Sig. | Partial η2 | PC  (mean±SD) | SM  (mean±SD) | MD  (mean±SD) |
| OffLine SS | 1.87 | .16 | .02 | 3.47±.11 | 3.43±.14 | 3.66±.07 |
| OffLine EI | 1.33 | .27 | .01 | 3.23±.13 | 3.23±.17 | 3.46±.09 |
| OffLine PSI | 1.14 | .32 | .01 | 3.46±.12 | 3.34±.16 | 3.59±.08 |
| OffLine AF | 1.16 | .32 | .01 | 3.74±.12 | 3.82±.16 | 3.96±.08 |
| OnLine SS | .51 | .60 | .01 | 2.07±.14 | 1.85±.18 | 1.96±.09 |
| OnLine EI | 1.98 | .14 | .02 | 1.94±.14 | 1.65±.18 | 2.05±.09 |
| OnLine PSI | .41 | .66 | .00 | 2.07±.15 | 1.86±.20 | 1.93±.10 |
| OnLine AF | 1.17 | .31 | .01 | 2.20±.17 | 2.03±.22 | 1.89±.11 |
| Rosenberg Self-Esteem | .35 | .71 | .00 | 22.35±.39 | 22.37±.51 | 22.70±.26 |
| Life Satisfaction | 1.83 | .16 | .02 | 4.80±.19 | 4.78±.25 | 5.18±.13 |

**Additional Table S1.** MANCOVA Results: Between subjects-effects of GROUP on OffLine scale and subscales, OnLine scale and subscales, Rosenberg Self-esteem scale and Life Satisfaction Scale. EI= Emotional and Informational; PSI=Positive Social Interaction; AF=Affectionate; SS=Social Support; PC=PC group (use the computer or tablet to surf the Internet); SP=SP group (use the smartphone to surf the Internet); MD= MD group (use more than one device to surf the Internet).
